# Initial Colony Morphology-Based Selection for iPS Cells Derived from Adult Fibroblasts Is Substantially Improved by Temporary UTF1-Based Selection

**DOI:** 10.1371/journal.pone.0009580

**Published:** 2010-03-08

**Authors:** Kurt Pfannkuche, Azra Fatima, Manoj K. Gupta, Rebecca Dieterich, Jürgen Hescheler

**Affiliations:** 1 Center of Physiology and Pathophysiology, Institute for Neurophysiology, University of Cologne, Cologne, Germany; 2 Center of Molecular Medicine Cologne, Cologne, Germany; Katholieke Universiteit Leuven, Belgium

## Abstract

**Background:**

Somatic cells can be reprogrammed into induced pluripotent stem (iPS) cells. Recently, selection of fully reprogrammed cells was achieved based on colony morphology reminiscent of embryonic stem (ES) cells. The maintenance of pluripotency was analysed.

**Methodology/Principal Findings:**

Clonal murine iPS cell line TiB7-4, which was derived from adult fibroblasts, was analysed for maintenance of pluripotency. Colony morphology, expression of pluripotency factors and stage specific embryonic antigen 1 (SSEA1) were analysed by real time PCR and flow cytometry. We found the iPS cell line TiB7-4 and its subclones to be rather diverse and exhibiting a tendency towards spontaneous differentiation and loss of pluripotency independent of their initial colony morphology. In contrast an undifferentiated transcription factor 1 (UTF1) promoter-driven G418 (Neo) resistance significantly improved the quality of these iPS cells. After selection with UTF-Neo for two weeks iPS subclones could be stably maintained for at least 40 passages in culture and differentiate into all three germ layers. As control, a construct expressing G418 resistance under the control of the ubiquitously active SV40 early promoter formed subclones with different colony morphology. Some of these subclones could be cultured for at least 12 passages without loosing their pluripotency, but loss of pluripotency eventually occured in an unpredictable manner and was independent of the subclones' initial morphology and SSEA1 expression. A UTF-Neo-based selection of a whole population of TiB7-4 without further subcloning resulted in the generation of cultures with up to 99% SSEA1 positive cells under stringent selection conditions.

**Conclusions:**

Our data indicate that temporary selection using a genetic UTF1-based system can generate homogenous pluripotent iPS cells that can be maintained without permanent selection pressure.

## Introduction

With the identification of defined factors enabling the reprogramming of somatic cells to an induced pluripotent state, a new area of stem cell biology has begun [1]. Initial reports identified a combination of the factors Oct4, Klf4, Sox2 and c-Myc to be sufficient to reprogram murine fibroblasts to a pluripotent state, albeit with very low efficiencies [1], [2]. The same combination of factors turned out to be sufficient to reprogram human fibroblasts [3], [4]. In addition Oct4, Sox2, Nanog and Lin28 were identified as an alternative combination, at least in human cells [5]. The use of small molecules as valproic acid and the finding that other cell types than fibroblasts are easier to reprogram have further improved the current method [6]–[8].

Novel approaches aim to reduce the number of factors needed for reprogramming and a reduction to two factors has been achieved [9], [10]. In murine adult neuronal stem cells, complete reprogramming could be achieved when only OCT-4 was expressed [11]. In addition, it has become possible to use adenoviral vectors to transduce somatic cells without integration of the viral vectors [12]. A recent approach by Thomson and co-workers utilized episomal vectors to introduce the reprogramming factors [13]. This elegant technique benefits from the graduate loss of the episomal vectors during prolonged cell culture resulting in iPS cells that are free of foreign DNA [13].

The efficiency of reprogramming is low and not all colonies that arise after reprogramming might represent fully reprogrammed cells, especially when they are derived from adult somatic cells [7]. Selection strategies have been applied with the aim to enrich pluripotent cells [2]. The selection of reprogrammed cells can be performed by the introduction of an antibiotics resistance controlled by a promoter specific for pluripotent cells. Promoters for Oct4 or Nanog were successfully used in this context [2]. It has been demonstrated recently with human embryonic stem cells, that G418 resistance driven by the *undifferentiated transcription factor1* (UTF1) promoter plus enhancer elements is very efficient to enrich the fraction of pluripotent cells within ES cell cultures [14]. UTF1 is expressed in embryonic carcinoma cells, embryonic stem cells and cells of the germ line but absent in adult tissues [15]. The *UTF1* promoter consists of a short TATA less region and a downstream enhancer at the 3-prime end of the coding sequence [15]–[17]. Functional binding sites for Oct4 and Sox2 were identified within this enhancer region. In addition, a genetic element called M1 could be found within the enhancer and carries an octamer sequence important for Nanog expression [14], [18]. Besides these elements, it is likely that additional, yet unknown factors contribute to the regulation of UTF1.

Experiments that demonstrate a fast downregulation of UTF1, which responds even faster to spontaneous and induced differentiation than Oct4 or Nanog, clearly point to a more complex regulation of the UTF1 expression [14], [16], [19], [20]. The fast response of the UTF1 expression upon induction of differentiation, which is probably supported by its low endogenous expression level, makes the *UTF1* promoter an interesting tool for the enrichment of high quality, homogenous pluripotent cell lines [14]. In this context, it is important to note that reprogramming of human fibroblasts demonstrated that UTF1 overexpression and a p53 knockdown act synergistically to enhance reprogramming efficiency of Oct4, Klf4, Sox2 and c-myc by 200-fold [21]. Knockdown of p53, which is believed to be suppressed by Klf4 or over-expression of UTF1 alone had a reduced supporting effects on reprogramming [21].

Recently, it has been reported that selection of fully reprogrammed cells can be based solely on colony morphology [22]. Reprogrammed cells that resemble the typical shape of embryonic stem cells could be expanded without the need of introducing selection cassettes for enrichment of pluripotent cells [22]. In this study, we investigated whether colony morphology is a sufficient criterion to identify high quality iPS cells that maintain their pluripotency in culture. In addition, we made use of an UTF1 promoter driven G418 cassette to select iPS cells for pluripotency and characterized the selected cells in comparison to iPS cells that were transfected with a G418 resistance marker driven by the ubiquitous SV40 early promoter.

## Results

### Characterization of the Morphology-Based Selected iPS Cell Line TiB7-4

**Figure 1:**
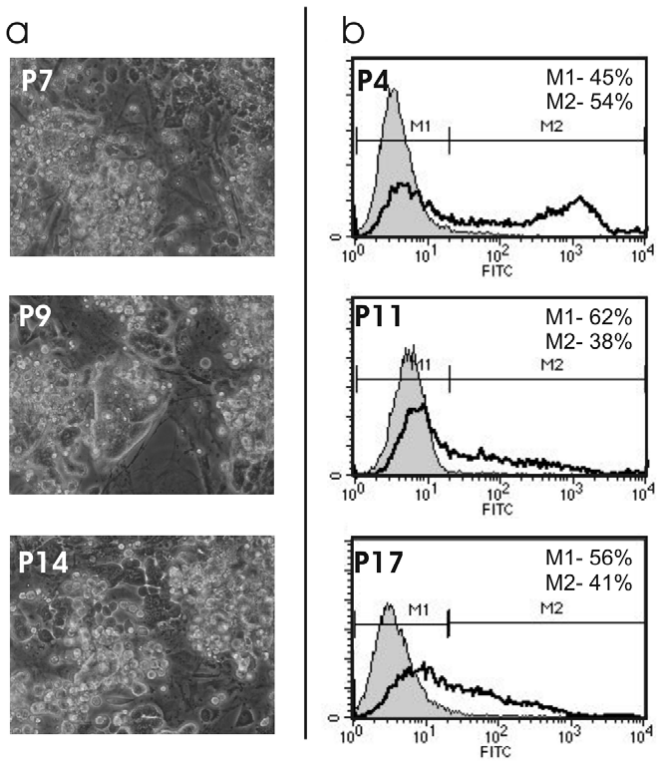
Morphology and SSEA 1 expression of wildtype TiB7-4 iPS cells. **a**) Morphology of TiB7-4 cultures at different passages of culture. **b**) Expression of stage specific embryonic antigen (SSEA1) at different passages of culture determined by flow cytometry.

Induced pluripotent stem cells grown from clone TiB7-4 [22] were cultivated on murine embryonic feeder cells in presence of LIF. The morphology of the colonies was studied at passage numbers 7, 9 and 14 (**Figure 1a**). A heterogenous morphology could be observed already at early passage numbers. Some colonies reminded of undifferentiated ES cells. Many colonies appeared fragmented at early passages and at higher passage numbers, compact colonies disappeared and the colonies broke up into single cells.

Stainings for stage specific embryonic antigen 1 (SSEA1) were performed at passages 4, 11 and 17 and analysed by flow cytometry (**Figure 1b**). The fraction of SSEA1 positive cells declined from 54% at passage 4 to 38% at passage 11 and 41% at passage 17. A strong reduction of the staining intensity for SSEA1 could be observed in passages 11 and 17 when compared to passage 4 (**Figure 1b**).

### UTF1-Neo Selection of Pluripotent iPS Subclones from TiB7-4

**Figure 2:**
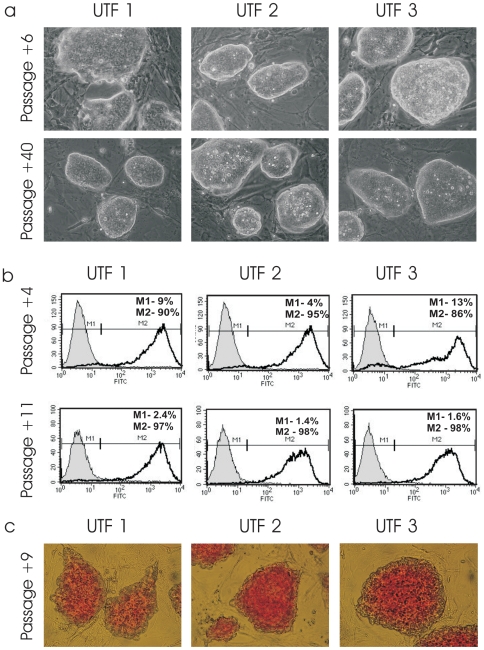
Characterization of three individual, UTF1-Neo selected iPS cell clones UTF-1, -2 and -3 derived from TiB7-4 cells. **a**) Morphology of UTF-Neo selected iPS cells six and forty passages after transfection. Selection with G418 was performed only until initial isolation of colonies. **b**) Expression of SSEA1 of UTF-1, -2 and –3 as indicated by flow cytometry four and eleven passages after transfection. **c**) Alkaline Phosphatase staining of UTF-1, -2 and –3.

To enrich pluripotent cells from the non-homogenous population of TiB7-4 cells, UTF1-Neo was used as selection vector. TiB7-4 cells were electroporated with circular UTF1-Neo plasmid and selected with G418 24 hours after the transfection, which ultimately resulted in several colonies. Three were isolated two weeks after electroporation and expanded as subclones UTF-1, UTF-2 and UTF-3. No further selection with G418 was applied. UTF-1, -2 and –3 were sub-cultured for 40 passages (**Figure 2a**) and, in contrast to unselected cells, formed homogenous colonies with elliptical shape and sharp borders (**Fig. 2a**). Stable integration of the UTF1-Neo vector was proven by PCR with primers specific for the UTF1 driven Neomycin resistance at passage 42 (**Figure S1**).

Expression of SSEA1 surface antigen was analysed by FACS at passages 4 and 11 after electroporation, as indicated by +4 and +11. At passage +4, a fraction of 90% SSEA1 positive cells was found in UTF-1, UTF-2 contained 95% of SSEA1 positive cells and UTF-3 86% (**Fig. 2b**). At passage +11, a fraction of 97% SSEA1 positive cells was found in UTF-1 and 98% in UTF-2 and UTF-3 cells (**Fig. 2b**). Staining for alkaline phosphatase indicated high levels of alkaline phosphatase in all three UTF-1 selected clones (**Fig. 2c**).

### Expression of Pluripotency Factors in UTF1-Selected iPS and Unselected TiB7-4

**Figure 3:**
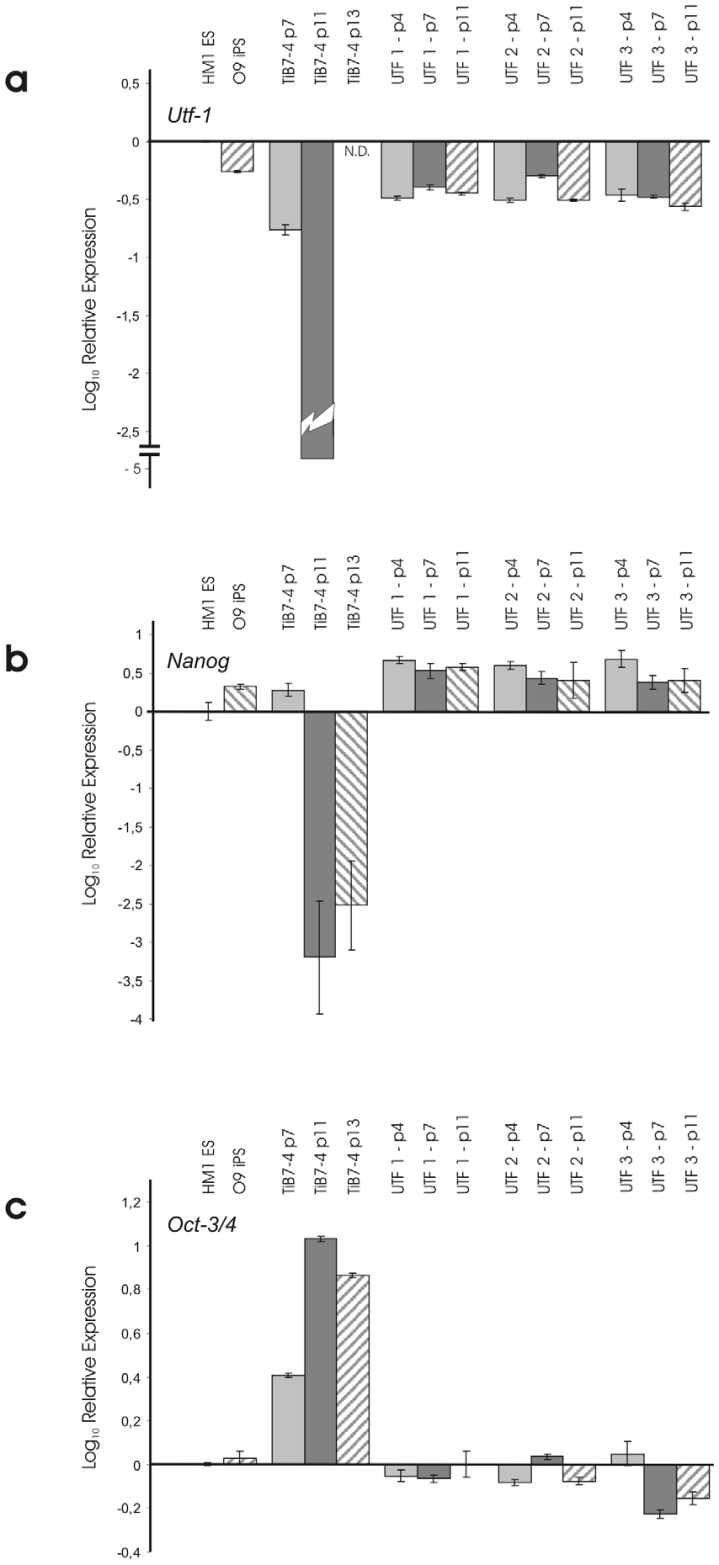
Comparison of relative expressions of Nanog (a), Oct-3/4 (b) and Utf-1 (c) in TiB7-4 wildtype iPS cells at different passages and UTF-Neo selected clones UTF-1, -2 and –3. Expression levels are normalized to HM-1 ES cells. The Oct-4-Neo selected iPS cell line O9 was included for comparison. N.D.: not detectable.

Expression of pluripotency factors UTF-1, Nanog and Oct4 was analysed by real time PCR (**Figure 3**). Parental TiB7-4 cells were included at passages 7, 11 and 13. UTF1-Neo selected subclones UTF-1, UTF-2 and UTF-3 were analysed at passages +4, +7 and +11. In addition cDNA from the mES cell line HM-1 was included in the study as well as cDNA from the Oct4-Neo selected iPS cell line O9. Quantification was normalized for HM-1 mES cells and revealed Utf1 expression levels of around 50% in O9 iPS cells. UTF-Neo selected cells range between 30% and 40% of endogenous UTF1 expression in most samples analysed (**Fig. 3a**). In contrast, expression of UTF1 could only be found at passage 7 in TiB7-4 wildtype iPS and drops to undetectable levels at later points.

Relative expression of Nanog was found to be two times higher in O9 iPS than in HM-1 mES cells and more than threefold higher in clone UTF-1. Clones UTF-2 and UTF-3 displayed Nanog expression levels that were at least twofold higher than Nanog expression in HM-1 (**Fig. 3b**).

Finally, expression of Oct4 shows a strong upregulation in parental TiB7-4 (**Fig. 3c**). From a more than twofold higher expression at passage 7, the Oct4 expression reaches tenfold higher values at passage 11 and slowly declines to around sevenfold higher expression at passage 13. To address the potential contribution of the reactivated viral *oct-4* gene used for reprogramming we performed real time PCR analysis to compare the expression of viral Oct4 in unselected TiB7-4 with clone UTF-1 and found the expression of viral Oct4 to be around 3500 time elevated in TiB7-4 with Ct values comparable to those of GAPDH (**Figure S2**).

### *In Vitro* Differentiation of UTF1-Selected iPS

**Figure 4:**
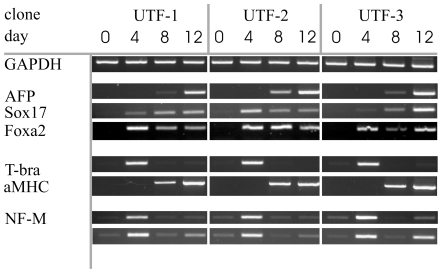
*In vitro* differentiation of UTF-Neo selected iPS clones UTF-1, -2 and –3. Embryoid bodies (EBs) were formed and cultured for up to twelve days. Cell samples were collected from undifferentiated cells as well as from EBs on day four, eight and twelve of differentiation and cDNA was prepared. Conventional reverse transcriptase PCR was used to test for marker gene expression indicative of formation of all three germlayers.

To analyse the capacity of the UTF1-selected iPS cells to differentiate towards all three germlayers, an *in vitro* differentiation assay was performed. Undifferentiated cells of UTF-1, UTF-2 and UTF-3 were cultured in suspension during permanent agitation to allow formation of embryoid bodies. Cell samples were collected on day 0 (undifferentiated cells) and on days 4, 8 and 12 of induced differentiation. Tissue samples were processed to extract total RNA and transcribed into cDNA. Polymerase chain reaction was applied to check for the presence of different key markers, using GAPDH as a housekeeping control gene (**Figure 4**). Alpha fetoprotein (AFP) and the transcription factors Foxa2 and Sox17 were included as markers for endodermal cells. AFP expression is detectable on day 8 and strongly expressed on day 12. Foxa2 is expressed at all timepoints analysed except in undifferentiated cells, Sox17 could be found in all differentiated samples with very low abundance at day 4 in UTF-3 cells. The cardiac-specific gene alpha myosin heavy chain marks the presence of cardiac cells that are of mesodermal origin and is present at day 8 and 12. Clone UTF-2 could be used to generate pure cardiomyocytes after introduction of a puromycin resistance under control of a cardiac specific promoter (data not shown). A progenitor population for mesoderm and some endodermal lineages is characterized by the expression of the t-box transcription factor t-brachyury, present on day 4 in all the three cell lines. Finally, ectodermal cell populations are indentified by the expression of neurofilament medium (NF-M) and fibroblast growth factor receptor 5 (Fgf5) with strongest expression on day 4 of differentation.

### Analysis of TiB7-4 Subclones Derived from TiB7-4 with SV40-Neo Selection

**Figure 5:**
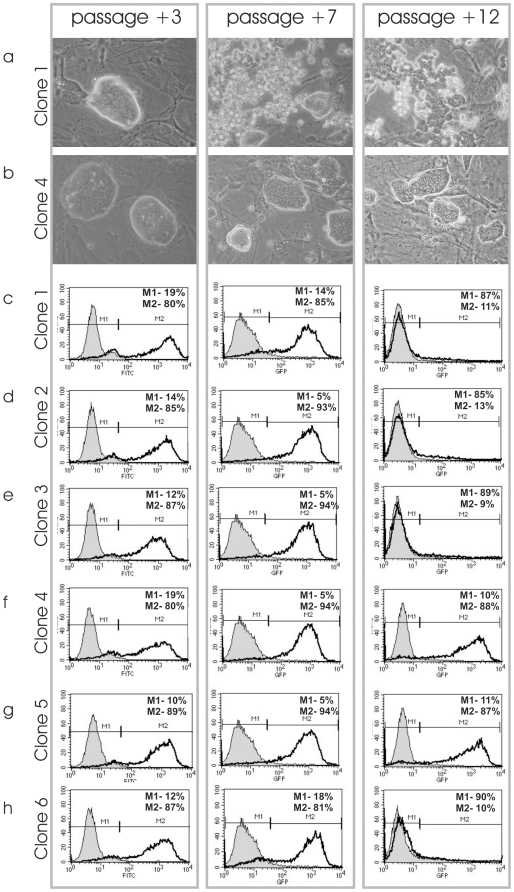
Morphology and SSEA 1 expression of SV40-Neo transfected control clones. Colony morphology of SV40-Neo selected clone 1 (a) and 4 (b) three, seven and twelve passages after transfection. SSEA1 expression of SV40-Neo selected clone 1-6 three, seven and twelve passages after transfection as determined by flow cytometry (c-h).

To rule out effects generated by electroporation or by selection for highly proliferative subclones, a control vector was transfected into TiB7-4 parental iPS cells. The SV40-Neo vector expresses resistance against G418 and is driven by the ubiquitously active early SV40 promoter. G418 selection then resulted in a variety of clones. In a first experiment, ten clones of the SV40-Neo transfected TiB7-4 were randomly selected and isolated. Morphology of these clones varied, ranging from ES cell-like to differentiated colonies (data not shown). Flow cytometric analysis of these clones at passage 3 resulted in different fractions of cells that were positive for the pluripotency marker SSEA1 (data not shown). In a second experiment, TiB7-4 cells were again transfected with SV40-Neo and subsequently selected with G418. Six clones with a typical morphology for mES cells were isolated and expanded. Stable integration of the SV40-Neo selection marker was proven by PCR amplification with primers for the SV40 promoter and the Neomycin resistance (**Figure S1**). The morphology of the colonies was analysed at passages +3, +7 and +12 after transfection (**Figure 5a, 5b**). At early passage, all six clones formed colonies that resembled the typical shape of mES cells but spontaneous differentiation set in after further passaging and resulted in a fragmentation of the colonies at passage +8 in the SV40-Neo transfected clone 1 (**Fig. 5a**) and clones 2, 3 and 6 (data not shown). The observed loss of regular colony morphology was even more pronounced at passage +12. Remarkably, there was no such change in colony morphology seen in clone 4 (**Fig. 5b**) and clone 5 (data not shown).

SSEA1 expression was analysed at passage number +3, +7 and +12 (**Fig. 5c–h**). All six clones show similar SSEA1 expression ranging from 80% to 89% at passage +3. At passage +7 even higher fractions of SSEA1 positive cells could be observed. At passage +12 clone 4 showed 88% and clone 5 87% SSEA1 positive cells, but the remaining four clones had almost lost SSEA1 expression (**Fig. 5c–h**).

### Expression of Pluripotency Factors in SV40-Neo Selected Subclones

**Figure 6:**
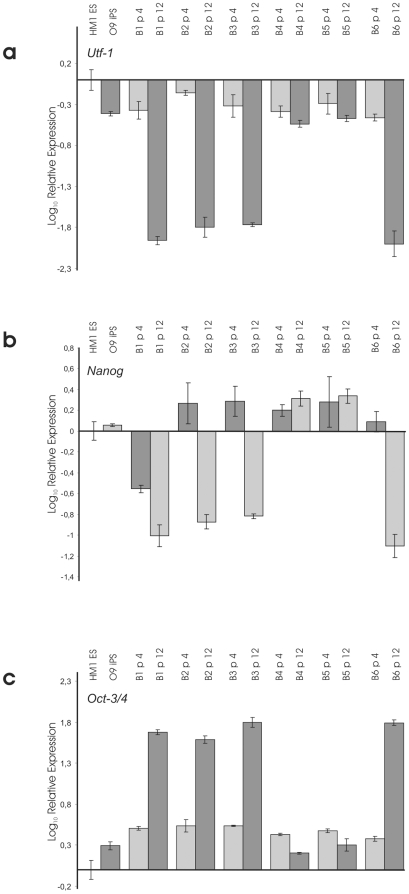
Comparison of relative expressions of Nanog (a), Oct-3/4 (b) and UTF-1 (c) in SV40-Neo transfected control clones 1 – 6 four and twelve passages after transfection. Expression levels are normalized to HM-1 ES cells. The Oct-4-Neo selected iPS cell line O9 was included for comparison.

Expression of pluripotency factors UTF1, Nanog and Oct4 was analysed by real time PCR. Samples collected at passage number 4 and 12 were included for all six SV40-Neo transfected and G418 selected clones. Normalization was done for expression levels in HM-1 ES cells. cDNA from O9 iPS cells was included for comparison. Analysis of expression levels, again normalized for HM1 ES cells, revealed a strong down-regulation of UTF1 and Nanog expression in SV40-Neo transfected clones 1, 2, 3 and 6 at passage 12 (**Figure 6a and 6c**). The expression of UTF1 was found to be comparable to O9 iPS cells at passage number 3 of all clones and at passage number 12 of clone 4 and 5 (**Fig. 6a**). Expression of Nanog was found to be stable and slightly enhanced at passage 4 and 12 of clone 4 and 5 (**Fig. 6b**). In contrast the expression of Oct4 showed strong upregulation at passage 12 of clones 1, 2, 3 and 6 (**Fig. 6c**).

### Non-Clonal Selection of a Pluripotent Cell Line from TiB7-4

**Figure 7:**
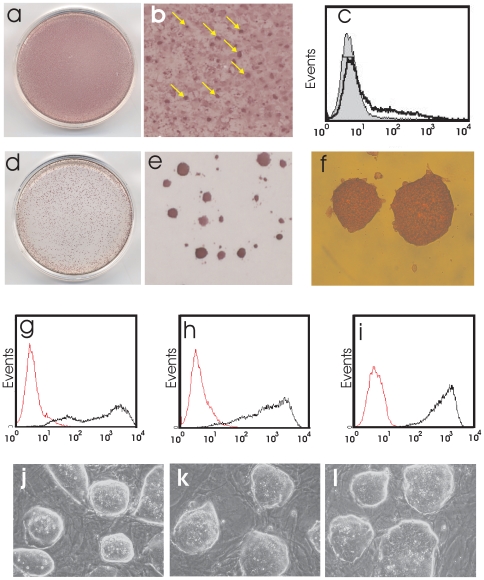
Non-clonal selection of iPS cells with UTF-Neo. Cultures of TiB7-4 iPS cells at passage 11 show flat growing cells with varying expressions of alkaline phosphatase (**a**) and contain clusters of cells that stain strongly for alkaline phosphatase (arrows) (**b**). When assayed for the expression of SSEA1 (**c**) the total amount of SSEA1 expressing cells was determined as 16% (black line). After transfection with UTF-Neo and selection on gelatine with 6 mg/ml G418 the flat growing cells vanish and sharp colonies with strong alkaline phosphatase (**d,e,f**) expression remain. Embryonic stem cells line Bruce4 (passage 26) displayed 84% SSEA1 positive cells (black line, **g**). UTF-Neo selected cells on feeder displayed 93% SSEA1 positive cells (**h**). UTF-Neo selected cells under continuous selection pressure on gelatine contained 99% SSEA1 positive cells with high expression of the surface marker (**i**). Colony morphology of Bruce4 ES cells (**j**) and UTF-Neo selected cells (**k,l**) were compared by phase contrast microscopy.

Experiments with TiB7-4 of more than 10 passages led to inefficient generation of stable pluripotent clones. Clonal colonies picked under 0,4 mg/ml G418 selection showed signs of spontaneous differentiation immediately following picking. A titration with G418 revealed a concentration of 6 mg/ml as a dose where no colonies with spontaneous differentiating cells could survive, attach or proliferate and led to a stabilization of the phenotype (data not shown). To apply this finding for improved and rapid generation of homogenous, stable pluripotent cell cultures even from low quality iPS cells we performed nucleofection of TiB7-4 with UTF-Neo. The initial TiB7-4 culture displayed heterogeneous alkaline phosphatase expression (**Fig. 7a**) with small clusters of cells exhibiting high expression of alkaline phospatase (**Fig. 7b**). The content of SSEA1 positive cells was analyzed by FACS and revealed 16% positive cells with a low expression density on most of the cells (**Fig. 7c**). After nucleofection a selection with 6 mg/ml G418 was applied for 10 days with a single passage of the cells during selection. After selection the culture formed round colonies with strong expression of alkaline phosphatase (**Fig. 7d–f**). Comparison of the SSEA1 expression with Bruce4 ES cells (passage 23) revealed a content of 83% positive cells for Bruce4 ES on feeder (**Fig. 7g**) and 93% SSEA1 for the UTF-Neo-selected on feeders (passage 4, **Fig. 7h**) and 99% for UTF-Neo-selected cells on gelatine under continued selection pressure (passage 3) (**Fig. 7i**). Comparison of the colony morphology of Bruce4 ES (**Fig. 7j**) and UTF-Neo selected non-clonal cells (**Fig. 7k,l**) revealed identical colony morphologies.

## Discussion

We have analysed in this study whether selection for iPS cells based on colony morphology is always suitable to generate high-quality cell lines, such as required for future transplantation studies and therapeutic applications. The iPS cell line TiB7-4 used for this study was generated by reprogramming of adult murine fibroblasts with the four transcription factors Oct4, Klf4, Sox2 and c-Myc [22]. The selection was performed based on colony morphology indicative for cells similar to murine ES cells. We found that this cell line alters its morphology within several passages, which is accompanied by a loss of SSEA1 expression and complete loss of Nanog and UTF1 expression. In contrast, a selection based on a human UTF1 promoter/enhancer driven neomycin-resistance transgene led to virtually absolute pluripotent cultures that could be maintained for at least 40 passages.

Alternative selection approaches utilized reactivation of the endogenous Nanog or Oct4 locus to indicate induction of pluripotency. While selection of reprogrammed fibroblasts with a Nanog-Neo construct was reported to result in a higher number of colonies compared to Oct4-Neo selection, only 5% of the resistant colonies with ES-like appearance revealed pluripotency but 22% of the Oct4-selected clones with ES-like appearance were found pluripotent [2]. Based on these report it can be assumed that the Nanog-driven selection is less suitable to generate high quality iPS cells. However the fraction of ES-like colonies among the G418-resistant colonies amounted to only 11.5% under Nanog-Neo selection and 24% under Oct4-Neo selection respectively.

A detailed characterization of a Oct4-Neo selected as well as a Nanog-Neo selected iPS cell line indicated a delay in the *in vitro* differentiation of both cell lines when compared to the ES lines R1 and D3 [23]. In the present study it could be shown that UTF-selection based iPS cells do not show a delay in differentiation. However further studies are needed to allow a final conclusion.

The establishment of homogenous cell populations is of importance for a variety of purposes. Lines of ES as well as iPS cells that contain a mixture of pluripotent and pre-differentiated cells might be less suited to perform *in vitro* differentiations to study aspects of differentiation or to form specific derivatives for detailed analysis. This is particularly true for human ES cell cultures that usually contain a subset of less pluripotent and pre-differentiated cells [24]–[26]. Gene expression profiling is one important technology to take a global view on gene expression and it might be suited to check novel iPS cell lines with respect of their similarity to ES cells with special focus on therapeutic applications. It is therefore highly desired to generate homogeneous populations of iPS cells since heterogeneous cultures will potential obscure findings.

Peter Droge and colleagues were able to demonstrate that an *UTF1* promoter driven Neomycin-gene is a sensitive selection marker in human ES cells to enrich pluripotent cells and to eliminate pre-differentiated cell types from the culture [14]. In addition, UTF1 is down-regulated faster than other pluripotency markers upon differentiation of ES cells [14]. For this reason, we hypothesised that UTF1-Neo selection might help to select for high-quality pluripotent cells that exists within heterogenous TiB7-4 iPS cell population. In fact, after transfection of TiB7-4 with UTF1-Neo and selection for two weeks with G418, three subclones of TiB7-4 were established. These clones displayed homogenous morphologies without signs of spontaneous differentiation for up to 40 passages indicating the presence of high-quality pluripotent cells within the TiB7-4 population.

Flow cytometric analysis for SSEA1 clearly indicated a substantial improvement of the culture after selection for UTF1-Neo with 97% to 98% SSEA1 positive cells in all three clones. With respect to the fact that the residual 2% of cells might represent feeder fibroblasts one can assume that all iPS cells in this culture are positive for SSEA1. Remarkably, this effect can be observed without continuous selection. We speculate that the complete removal of pre-differentiated cells leads to a terminal stabilization of the UTF1-Neo selected clones. While pre-differentiated cells might influence the cell signalling and induce differentiation of neighbouring cells, populations that are depleted of pre-differentiated cells might exhibit a more stable pluripotent phenotype. The described selection, however, does not influence the ability of the UTF1-Neo selected cells to form EBs and to undergo differentiation within these spheroids giving rise to all three germlayers, which was also demonstrated earlier for UTF1-selected hES lines [14].

It is worth mentioning that the most stringent assay applied in the present study is the *in vitro* differentiation assay that corresponds to the teratoma-formation assay *in vivo*. As most stringent assays in murine systems chimaera formation and tetraploid complementation are commonly accepted. For human cells the teratoma assay and *in vitro* differentiation assay are the most stringent pluripotency assays applicable.

Interestingly, expression of UTF1 and Nanog declined very soon and dropped to undetectable levels at passage 13 in parental TiB7-4 cells while expression of Oct4 is up to tenfold upregulated compared to HM-1 ES cells. The current data gives the impression that the overshooting Oct4 expression is correlated with the strong spontaneous differentiation on passage 11 and declines afterwards, but this observation needs further analysis. Earlier reports link elevated levels of Oct4 expression to endodermal differentiation of ES cells [27], [28]. This might point to a preferentially endodermal fate when spontaneous differentiation sets on in non-selected TIB7-4 cells. A reactivation of the viral Oct4 transgene could be shown by quantitative PCR comparing the expression of viral Oct4 in the selected UTF-1 clone at different passages with the expression in TiB7-4 iPS cells at different passages. The expression of viral Oct4 was found to be 3 orders of magnitude higher in unselected cells. The expression of retroviral Oct4 found in this study is in line with the pioneer study of Wernig and colleagues who describe a silencing of retroviral vectors after reprogramming with residual expression of the retroviral reprogramming factors [2].

Proper silencing of viral reprogramming factors is believed to be associated with the formation of stable iPS cultures and this hypothesis is in line wit our finding that the expression of viral Oct4 is much lower in the selected clone UTF1 [29]. However a later reactivation of the viral transgenes might lead to impaired stability of the pluripotent phenotype and this concern would point to the need to use reprogramming strategies that do not utilize retroviral vectors that are maintained in the genome.

Control transfections with a G418 resistance under the control of the ubiquitous active SV40 early promoter let to the identification of cell clones that partially resembled a perfect ES cell like elliptical colony shape with sharp borders. Closer analysis of six control clones resulted in the surprising finding that four out of these six clones spontaneously underwent differentiation after some passages what was first obvious by disturbed colony formation and downregulation of UTF1 and Nanog and upregulation of Oct4, and later by a loss of SSEA1 surface antigen. The remaining two clones, however, seemed to be stable pluripotent at least during the observation period of 12 passages. The reason for the instability is not clear and it might be speculated that it results from incomplete reprogramming or reactivation of the reprogramming virus.

The nucleofection of low quality starting material with UTF-Neo resulted in the generation of a pluripotent cell line within 10 days that could readily be expanded and used for experiments. The elevated selection pressure used in this experiment had turned out to separate plutipotent cells from predifferentiated cells in pilot experiments and we suggest estimating the level of G418 individually for iPS cells generated from different sources.

The discussed findings underline that a selection of iPS cells based on colony morphology is not sufficient to prove the fully reprogrammed state of the cells and a stable pluripotent phenotyp. Fully reprogrammed cells within an iPS colony can be effectively isolated by selection with UTF1-Neo. Alternatively a selection based on the colony morphology might be suitable but requires the culture of the cells over a prolonged time and the surveillance of the pluripotency markers Nanog and UTF1 by real time PCR. Further studies will address the duration of UTF1-Neo based selection needed to achieve homogenous pluripotent cells. It can be speculated that shorter selection intervals than the two weeks employed here might be sufficient to obtain UTF1 selected cultures, perhaps even without stable integration of the selection cassette into the iPS genome. The application of methods like transient nucleofection, adenoviral or baculoviral transduction to transiently introduce the UTF1-Neo vector into the iPS cell population might ultimately combine the benefit of selection with the advantage of transient transgene delivery. This might become especially relevant for the generation of iPS from adult human somatic cells for clinical applications, since these cells appear to be more refractory to reprogramming than other cell types, such as adult stem or progenitor cells.

## Materials and Methods

### Cell Lines and Cell Culture

The iPS cell lines TiB7-4 and O9 were generated by Alexander Meissner and Marius Wernig at the laboratory of Rudolf Jaenisch at the Whitehead Institute of Technology, MA, USA [2], [22]. Cells were cultured in ES medium on inactive murine embryonic fibroblasts cells (MEF). MEFs were grown from HIMOF1 outbred mice at embryonic day 14.5 and mitotically inactivated by mitomycin C (Serva Electrophoresis GmbH, Heidelberg, Germany). ES medium consisted of Iscoves modified Dulbeccos medium (IMDM) supplemented with 17% fetal calf serum (FCS, Invitrogen GmbH, Karlsruhe, Germany), 1% non/essential amino acids (Invitrogen), 100 units/ml Penicillin and 100 µg/ml Streptomycin (Invitrogen), 100 µm β-mercaptoethanol (Sigma-Aldrich, Germany) and 1000 units/ml leukaemia inhibitory factor (ESGRO, Millipore, Schwalbach, Germany).

### Plasmids, Electroporation and Nucleofection of iPS Cells

The UTF1-Neo construct has been described before [14]. SV40-Neo was generated from pHcRed1.1 (Clontech-Takara Bio Europe, Saint Germain en Laye, France). The coding sequence for HcRed was removed by digest with AgeI and NotI. The remaining vector backbone holds a resistance again G418 (neomycin-resistance gene) under the control of the ubiquitously active SV40 early promoter. Electroporation employed circular plasmids. Briefly, 5–10×10^6^ cells were resuspended in PBS containing 40 µg plasmid and electroporated at 260 volts and 500 µF using a GenePulser instrument (Bio-Rad Laboratories, Munich, Germany). After electroporation, cells were plated on G418 resistant MEFs. Selection was with 400 µg/ml G418 (Invitrogen) and started 24 hours following electroporation.

Nucleofection was performed using the Nuclefector II device (Lonza) with the murine embryonic stem cell nucleofection kit. Program A024 was used to nucleofect 6 µg DNA according to manufacturers instructions. Selection was started 4 hours after nucleofection.

### SSEA1 Stainings and Flow Cytometry

Stage Specific Embryonic Antigen-1 (SSEA1) is a typical surface marker to determine the pluripotent state of murine ES cells. Cell-surface-antigen expression of iPS cells was assessed by indirect immunofluorescence and detected by flow cytometry. Single cell suspension of iPS cells was prepared by trypsinization. Cell clumps were removed by passing through the cell strainer (BD Falcon®) and collected into a falcon tube. A pellet of app. 0.5×10^6^ cells was resuspended in 50 µl of 1% FBS in PBS. Cells were then incubated with 1∶50 dilution of primary antibody anti-mouse SSEA1 (480, mouse monoclonal, Cat No. sc-21702, Santa Cruz) at 4°C for 30 minutes. A second set of tubes was also set using an isotype control primary mouse monoclonal IgM antibody (Cat no. sc-3881, Santa Cruz). Later the cells were washed twice with PBS to remove unbound antibody and finally stained with 50 µl of Fluorescein isothiocyanate (FITC)-labeled goat anti-mouse IgM secondary antibody for 30 minutes. The secondary antibody was used at a dilution of 1∶200. Finally the cell suspension was analyzed on a flow cytometer FACScan (BD Pharmingen) using CellQuest software (BD Pharmingen). Dead cells were excluded by gating on viable cells on the basis of staining with propidium iodide (Sigma). At least 10,000 live (PI negative) events were acquired for each sample.

### Alkaline Phosphatase Staining

For alkaline phosphatase stainings cells were fixed with 100% methanol and air-dried. Napthol AS-MX (Sigma-Aldrich) was dissolved in 0.1M Tris pH9.2 at a concentration of 2 mg/ml. Fast Red TR saltTM (Sigma-Aldrich) was dissolved at a concentration of 1 mg/ml in 0.1M Tris pH 9.2. All solutions were prepared fresh. The Napthol solution was added to a final concentration of 200 µg/ml to the Fast Red TR salt-solution. Cells were incubated for 15 minutes at 37°C. To stop the reaction, cells were rinsed well with PBS. Alternatively a commercially available alkaline phosphatase staining kit (Millipore) was used according to manufacturers instructions.

### Preparation of cDNA and Reverse Transcriptase Polymerase Chain Reaction (RT-PCR)

For reverse transcriptase polymerase chain reactions undifferentiated cells or embryoid bodies were collected in a small centrifuge tube. Cells were dissociated in Trizol reagent (Invitrogen) and total RNA was isolated following manufacturers instructions. 2 µg of total RNA were treated with DNAseI (amplification grade, Invitrogen) and 750 ng of DNAse treated RNA were used for reverse transcription. cDNA synthesis with Superscript II Reverse transcriptase (Invitrogen) was primed using random hexamers. Generation of cDNA was performed in a total reaction volume of 20 µl. Finally the cDNA was filled up to 120 µl with nuclease free water. To analyse expression of key marker genes PCR reactions were performed in 30 µl total reaction volume using JumpStart RedTaq ReadyMix (Sigma-Aldrich) and specific primers as listed is table 1.

**Table 1:** Primers used for RT-PCR and proof of transgene integration.

gene	Foreward primer	Reverse primer
GAPDH	GTGTTCCTACCCCCAATGTG	CTTGCTCAGTGTCCTTGCTG
qGAPDH	GGCTCATGACCACAGTCCAT	ACCTTGCCCACAGCCTTG
AFP	CCTATGCCCCTCCCCCATTC	CTCACACCAAAGCGTCAACACATT
t-bra	CATGTACTCTTTCTTGCTGG	GGTCTCGGGAAAGCAGTGGC
αMHC	ACCTGGGCAAGTCTAACAAC	CTGGATTCTGGTGATGATACG
NFM	TAGAGCGCAAAGATTACCTGAAG	TTGACGTTAAGGAGATCCTGGTA
Sox17	TAAAGGTGAAAGGCGAGGTG	GCTTCTCTGCCAAGGTCAAC
Fgf5	GCTGTGTCTCAGGGGATTGT	TCTTGGCTTTCCCTCTCTTG
Foxa2	ACACGCCAAACCTCCCTACT	GGCACCTTGAGAAAGCAGTC
RV-Oct4	TACACCCTAAGCCTCCGCCT	ATTCCGGCGCCTAGAGAAG
UTFNeo	GGGTACCCCATGATTGAACA	CAGGTCGGTCTTGACAAAAAG
SV40Neo	CATTCTCCGCCCCATGGCTGAC	CCATGATGGATACTTTCTCG

To study integration of transgenes specific primer pairs UTFNeo and SV40Neo were used according to table 1. After initial denaturation at 94°C for 2 minutes all amplifications were carried out using denaturation at 94°C for 35 seconds, annealing at 56°C for 45 seconds, and elongation at 72°C for 75 seconds. After 35 cycles (32 cycles for GAPDH) the final elongation was carried out at 72°C for 5 minutes. PCR fragments were resolved on 2% agarose (GTQ agarose, Carl Roth GmbH+Co KG, Karlsruhe, Germany) gels containing ethidium bromide.

### Real Time PCR

For real time quantitative PCR measurements cDNA was prepared as described above. The cDNA derived from the reverse transcription was initially diluted to a total volume of 120 µl. A fraction of the cDNA was further diluted at a ration of 1∶20 in nuclease free water (Invitrogen). QuantiTect SYBR green PCR kit (Qiagen, Hilden, Germany) was used for the PCR reaction. For each single reaction, 10 µl of the 2x SYBR green stock solution were used and 2 µl of the diluted cDNA were added. Primer sets for Oct-3/4, Utf1 and Nanog were purchased from Qiagen (QuantiTect Primer Assay Kits) and added according to manufacturer's protocols. Primers for GAPDH (primers qGAPDH, see table 1) were used as standards. Expression of viral Oct4 was measured with primers RV-Oct4 (table 1). Nuclease-free water was added to a final volume of 20 µl. Reactions were performed in 96-well real time PCR reaction plates (AppliedBiosystems, Foster City, CA, USA) and sealed with adhesive films (AppliedBiosystems). Cycling was done in a Fast System 7500 Real Time PCR Cycler (Applied Biosystems) according to the following program: step 1: 10 minutes at 95°C; step 2: 15 seconds at 95°C; step 3: 30 seconds at 55°C; step 4: 45 seconds at 60°C (data collection was performed at this step); steps 2 to 4 where repeated for 40 cycles.

## Supporting Information

Figure S1 Stable insertion of selection markers. Integration of SV40-Neo was probes with primers specific for the SV40 promoter and the Neomycin resistance (a). Primers for the UTF1-promoter driven Neomycin resistance were used to test for stable integration of the UTF1-Neo transgene (b).
(1.29 MB TIF)

Figure S2 Expression of viral Oct4 in UTF-Neo-selected and non-selected cells. a) Relative expressions of the viral *oct-4* gene were assayed by real time PCR. Normalized for the expression of viral Oct4 in the selected clone UTF-1 passage 4 the relative expression level of viral Oct4 are shown for later passages (7, 11) of UTF-1 and for non-selected TiB7-4 iPS cells. Relative expression levels are shown in logarithmic scale. b) Average Ct-values of the real time PCR measurement are shown for all quantitative PCR assays depicted in (a).
(0.38 MB TIF)
