# Supplementary figures and images for: Initial Colony Morphology-Based Selection for iPS Cells Derived from Adult Fibroblasts Is Substantially Improved by Temporary UTF1-Based Selection
Source: PLoS One. 2010 Mar 8;5(3):e9580. doi: 10.1371/journal.pone.0009580 (PMC2833193; doi:10.1371/journal.pone.0009580)

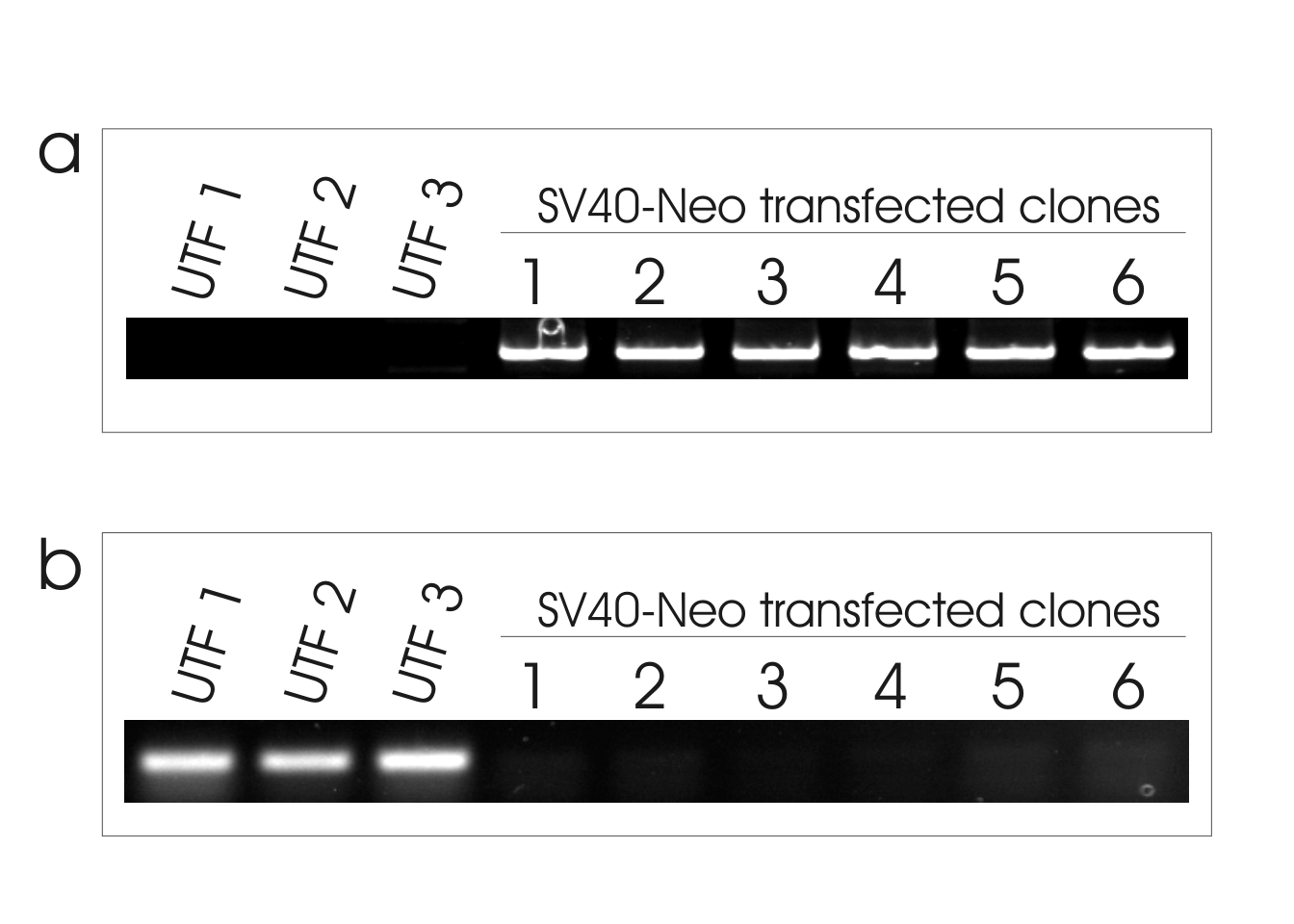

Supplement: Figure S1 — Stable insertion of selection markers. Integration of SV40-Neo was probes with primers specific for the SV40 promoter and the Neomycin resistance (a). Primers for the UTF1-promoter driven Neomycin resistance were used to test for stable integration of the UTF1-Neo transgene (b). (1.29 MB TIF) [file pone.0009580.s001.tif]

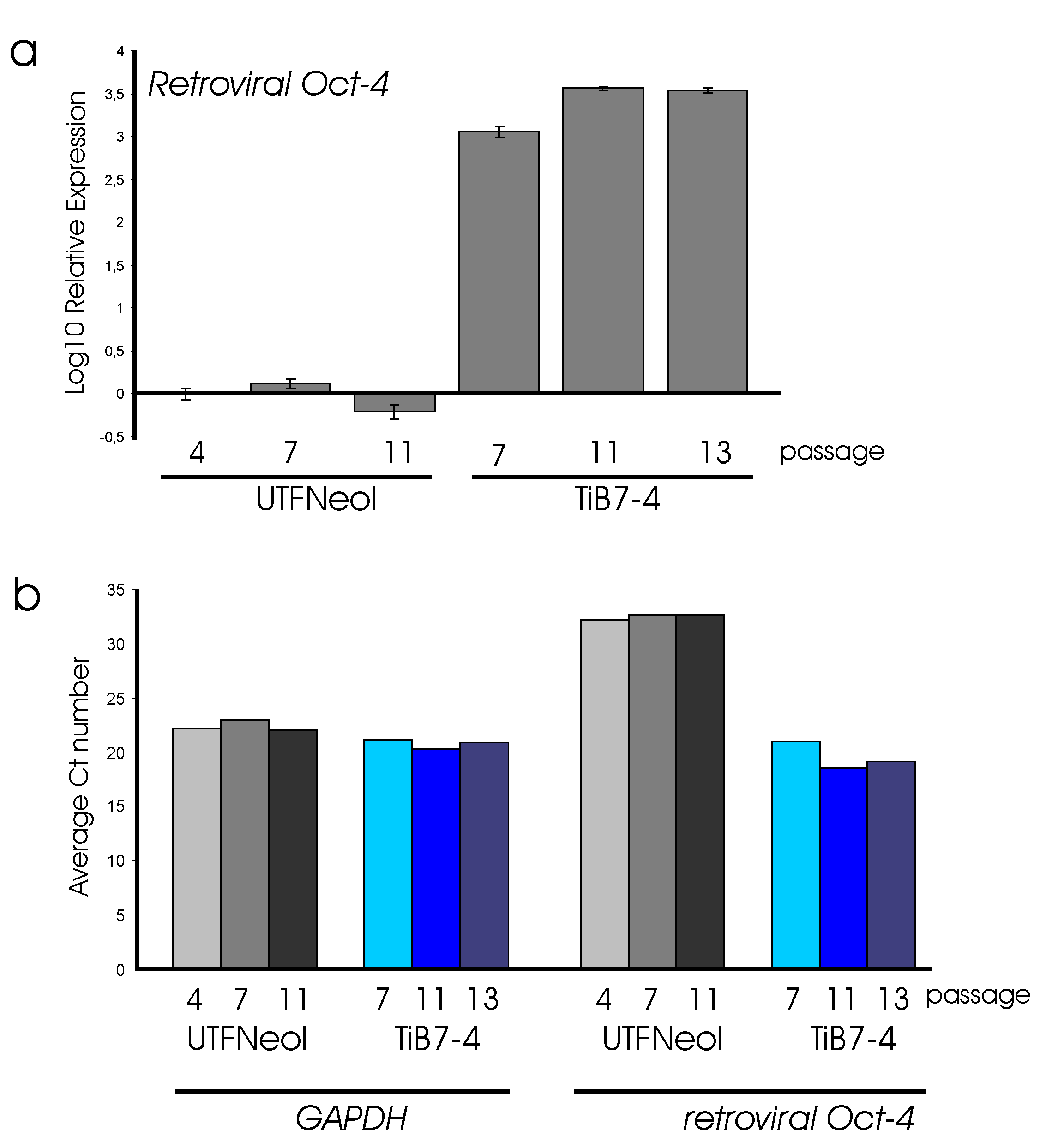

Supplement: Figure S2 — Expression of viral Oct4 in UTF-Neo-selected and non-selected cells. a) Relative expressions of the viral oct-4 gene were assayed by real time PCR. Normalized for the expression of viral Oct4 in the selected clone UTF-1 passage 4 the relative expression level of viral Oct4 are shown for later passages (7, 11) of UTF-1 and for non-selected TiB7-4 iPS cells. Relative expression levels are shown in logarithmic scale. b) Average Ct-values of the real time PCR measurement are shown for all quantitative PCR assays depicted in (a). (0.38 MB TIF) [file pone.0009580.s002.tif]
